# First Annual Meeting of the American Medical Association

**Published:** 1848

**Authors:** 


ARTICLE I

### FIRST ANNUAL MEETING OF THE AMERICAN MEDICAL ASSOCIATION.

As our colleague, Dr. Herrick, was detained in traveling East, so as not to reach there in time to attend the meeting of this association at Baltimore in May, we are unable to furnish our readers with an original account of its doings, and can only give an abstract from the notices published in our exchanges, the fullest of which is in the Medical News.

The Association assembled on Tuesday, the 2d of May The President, Dr. Chapman of Philadelphia, made an appropriate address. The day was spent in examining credentials of delegates, and making other preliminary arrangements. About 200 members were present at the opening; the number was increased by the arrival of others before the close of the session, to 256.

On Wednesday, by adopting the report of a committee of one member from each State represented, the following officers were unanimously elected for the ensuing year:

*President*—Dr. A. H. Stevens, of New-York.

*Vice Presidents*—Drs. J. C. Warren, of Mass., Sam.'l Jackson, of Penn., Paul F. Eve, of Ga., and Wm. M. Awl, of Ohio.

*Secretaries*—Drs. A. Stille, of Phil'a, and H. J. Bowditch, of Boston.

*Treasurer*—Dr. Isaac Hays, of Phil'a.

The subject of the importation, of adulterated and worthless drugs, occupied a considerable portion of the attention of the Association during the forenoon. Dr. T. O. Edwards, member of Congress from Lancaster, Ohio, was introduced, and delivered an address on the subject. His report to the House of Representatives as chairman of a special committee on the same subject, is noticed on another page of this number of the Journal.

Reports from the standing committee on surgery were made by Drs. Norris and Parish, and from the committee on obstetrics by Dr. H. Lindsley, all of which were referred to the committee on publication.

The afternoon session raised a committee of one from each State to nominate the standing committees for the present year.

Dr. O. W. Holmes made a report as chairman of the committee on medical literature. It was also referred to the committee on publication.

On the subject of the employment of anæsthetic agents' taken up as a part of the reports on surgery and obstetrics, the convention took no action, but referred it to the appropriate committees.

Thursday morning, some resolutions offered by Dr. Cohen, of Baltimore, complimentary to the medical officers of our army and navy were passed.

The committe previously appointed for the purpose, reported names for standing committees which were appointed. We have room only to enumerate the chairmen—they are as follows:

*Committee of Arrangements*—Dr. J. Biglow, of Boston.

*On Medical Sciences*—Dr. L. P. Yandell, of Ky.

*On Practice of Medicine*—Dr. Codie, of Penn.

*On Surgery*—Dr. N. R. Smith, of Md.

*On Obstetrics*—Dr. Wellford, of Va.

*On Medical Literature*—Dr. J. P. Harrison, of Ohio.

*On Medical Education*—Dr. F. C. Stewart, of N. Y.

*On Publication*—Dr. I. Hays, of Phila.

The committee being instructed to recommend a place for the next meeting, proposed Boston; which was agreed to.

Dr. Wellford presented the report of the committee on medical education, with a series of resolutions which, after amendment, were adopted. The first of these is as follows:

*Resolved*, That this association considers defective and erroneous every system of medical instruction, which does not rest on the basis of practical demonstration, and clinical teaching, and that it is therefore the duty of the medical schools to resort to every honorable means to obtain access for their students to the wards of a well-regulated hospital.

The second recommends trustees to open hospitals to students. The third condemns political influences in selecting physicians to hospitals. The fourth urges the standard of preliminary education, and requisites for graduation, fixed by the meeting in Philadelphia, The fifth requests faculties of medical schools, to make daily or weekly recapitulatory examinations of their lectures, and to adopt means of ascertaining the attendence of students to the close of the term. The sixth recommends that the final examination for the degree be made in presence of official judges of their qualifications, who are not interested pecuniarily. The seventh recommends that students be required to report cases for their inaugural theses, or in addition to them. And the eighth recommends that schools furnish their statistics to the chairman of the committee on medical education annually.

On motion by Dr. J. L. Atlee, the physicians of those States in which no State medical societies exist, were recommended to organize them.

The committee on the communication of the National Institute, in reference to hygiene, reported and recommended the appointment of a committee on hygiene of 12 members, which was agreed to, and the committee appointed, of which Dr. Wynne of Md. is chairman.

Friday morning, Drs. Geo. B. Wood of Phila., Jacob Biglow of Boston, and H. H. McGuire of Winchester, Va., were appointed delegates of the Association to the meetings of the British Association, and the Provincial Med. and Surg. Association.

It was decided that copies of the proceedings be sent to members who pay the assessment of three dollars for defraying expenses. Several propositions to amend the constitution, by rule lie over one year.

After various votes of thanks and instructions to committees and members, the Association adjourned *sine die.*

The meeting was characterized by professional devotion, harmony and good feeling, which we trust by its influence will be speedily diffused through the whole profession. [E.

